# Ostéoblastome de l’os pariétal de la voûte du crâne: à propos d’un cas

**DOI:** 10.11604/pamj.2020.36.160.16031

**Published:** 2020-07-07

**Authors:** Zoubida Bargach, Abderrazak Bertal, Idriss El Fathi, Khadija Ibahioin, Abdelhakim Lakhdar

**Affiliations:** 1 Service de Neurochirurgie, Centre Hospitalier Universitaire Ibn Rochd, Casablanca, Maroc

**Keywords:** Ostéoblastome, os pariétal, voûte du crâne, tumeur osseuse, anatomo-pathologie, Osteoblastoma, parietal bone, cranial vault, bone tumor, anatomopathological

## Abstract

L´ostéoblastome est une tumeur osseuse primitive peu fréquente, sa localisation au niveau de la voûte du crâne est extrêmement rare. Nous rapportons un cas d´ostéoblastome de l´os pariétal droit chez une femme âgée de 46 ans ayant des antécédents de traumatisme crânien bénin. Elle a présenté de façon progressive une tuméfaction pariétale droite douloureuse non inflammatoire. Le scanner crânio-cérébral a montré une lésion osseuse hyperdense respectant la table interne de l´os pariétal droit. La patiente a bénéficié d´abord d´une biopsie puis d´une résection totale de la lésion osseuse avec une cranioplastie au ciment méthyl-méthacrylique. Les suites post-opératoires étaient simples. L´examen anatomo-pathologique a montré qu´il s´agissait d´un ostéoblastome sans signes de malignité. Nous discuterons, à travers notre premier cas d´ostéoblastome de la voûte du crâne et une revue de la littérature, sa présentation clinique, l´examen anatomo-pathologique, les aspects radiologiques, ainsi que la prise en charge de cette rare pathologie.

## Abstract (en)

Osteoblastoma is an uncommon primary bone tumor. Its occurrence in the cranial vault is extremely rare. We here report our first case of right parietal bone osteoblastoma in a 46-year old woman with a history of benign cranial traumas. She reported progressive painful, non-inflammatory right parietal bone swelling. Craniocerebral CT scan showed hyperdense bone lesion with sparing of the internal table of the right parietal bone. The patient first underwent biopsy, then complete resection of the bone lesion with methyl-methacrylic cement cranioplasty. The postoperative course was uneventful. Anatomopathological examination showed osteoblastoma with no sign of malignancy. This study and literature review highlight the clinical manifestation, the radiological and anatomopathological features as well as the management of osteoblastoma of the parietal bone of the cranial vault.

## Introduction

L´ostéoblastome est une tumeur osseuse rare, elle représente 1% des tumeurs primitives osseuses et affecte généralement le rachis, les os longs, les os de la face et la mandibule [1,2]. La localisation au niveau de la voûte du crâne est extrêmement rare [1,3]. Depuis sa première description rapportée en 1956 par Jaffe et Lichtenstein [4], seuls quelques rares cas ont été décrits dans la littérature. Nous rapportons notre premier cas d´ostéoblastome de l´os pariétal de la voûte du crâne, ainsi qu´une revue de la littérature.

## Patient et observation

**Figure 1:**
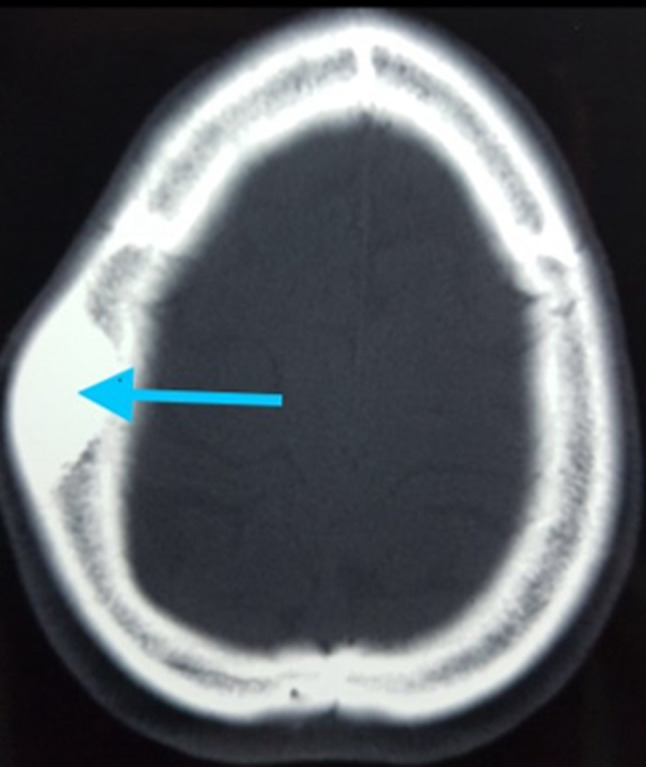
TDM cérébrale en fenêtre osseuse montant la lésion pariétale hyperdense sans lyse osseuse ni envahissement du parenchyme cérébral

Il s´agit d´une patiente de 46 ans, femme au foyer et sans antécédents pathologiques particuliers. Elle se plaignait d´une tuméfaction de la région pariétale droite de la voûte du crâne légèrement douloureuse et augmentant progressivement de volume pour laquelle elle n´a jamais consulté. Elle s´est présentée initialement au service des urgences à la suite d´un accident de la voie publique ayant occasionné un traumatisme crânien fermé, avec une légère perte de connaissance, sans vomissements ni convulsions ni autres points d´impact. A son admission, la patiente était consciente Glasgow à 15/15, sans déficit focal, L´examen neurologique était strictement normal. La palpation du crâne retrouvait une masse pariétale droite, légèrement douloureuse, de consistance dure, fixe par rapport au plan osseux, et sans signes inflammatoires en regard. Un scanner crânio-cérébral avait alors été demandé, aucune lésion traumatique n´a été retrouvée, cependant un épaississement hyperdense de l´os pariétal droit avait été mis en évidence sur les coupes en fenêtre osseuse, sans signe de destruction de périoste ni d´invasion du parenchyme cérébral, cette lésion respectait la table interne de l´os évoquant une tumeur osseuse bénigne (Figure 1).

**Figure 2:**
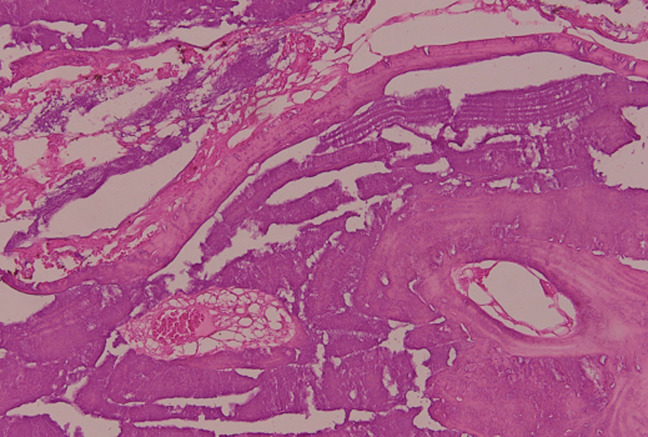
aspect histologique montrant des travées osseuses épaisses entrelacées; le stroma est peu abondant et montre une richesse en vaisseaux congestifs; hématéine-éosine, grossissement x 20

**Figure 3:**
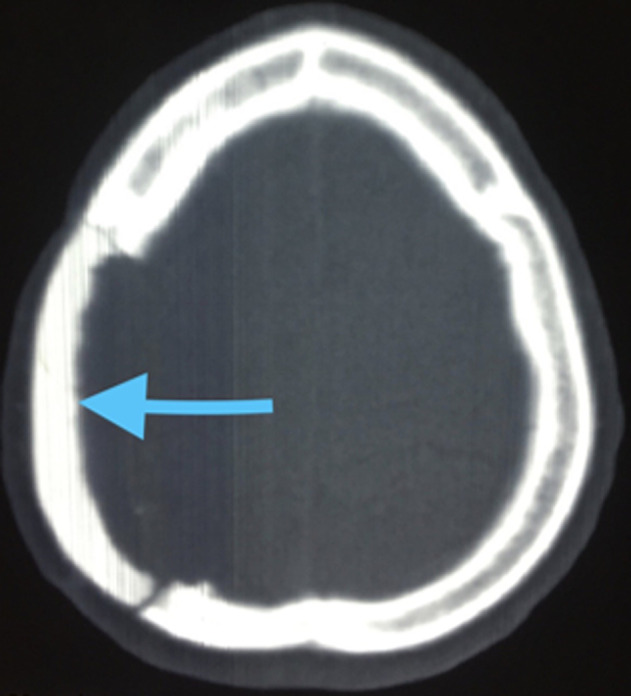
TDM cérébrale en fenêtre osseuse après cranioplastie

L´IRM crânio-cérébrale n´a pas été réalisée. La patiente a bénéficié d´abord d´une biopsie exérèse qui a confirmé le diagnostic d´ostéoblastome, elle a ensuite été opérée à travers une incision arciforme pariétale droite, après rugination de la galéa et du muscle temporal, un volet osseux dépassant légèrement la tumeur passant ainsi en os sain a été réalisé, ce qui a permis une ablation totale et en bloc de celle-ci. La dure-mère était intacte, le défect osseux résiduel a été comblé par une crânioplastie au ciment chirurgical à la méthyl-méthacrylique. Les suites post-opératoires immédiates étaient simples. L´examen anatomo-pathologique définitif a révélé un réseau de travées osseuses lamellaires épaisses, entrelacées, autour de nombreuses cavités vasculaires, concluant qu´il s´agissait d´un aspect morphologique d´un ostéoblastome (Figure 2). L´évolution à moyen terme était favorable avec disparition des douleurs, une bonne cicatrisation cutanée, sans signes d´infection ni de récidive tumorale (Figure 3).

## Discussion

L´ostéoblastome est une tumeur osseuse primitive rare [1,3,5]. Elle représente 1% des tumeurs osseuses, sa première description a été rapportée par Jaffé et Mayer qui ont publié en 1932, cependant ce premier cas d´ostéoblastome a été d´abord confondu avec un ostéome ostéoïde, et c´est Lichtenstein qui en 1956, a isolé l´ostéoblastome comme entité à part [4]. Néanmoins, malgré cette distinction entre ostéome ostéoïde et ostéoblastome qui vient des caractéristiques épidémiologiques, radio-cliniques, évolutives et surtout histologiques, il reste un diagnostic différentiel parfois difficile. La taille de la tumeur est un critère important pour le diagnostic. L´ostéome ostéoïde est évoqué lorsqu´elle est inférieure à 2 cm de diamètre, alors que l´ostéoblastome a une croissance rapide et sa taille est souvent supérieure à 3 cm [6]. L´étiopathogénie n´est pas claire et reste largement débattue dans la littérature. L´ostéoblastome affecte généralement la colonne vertébrale (40%) et les os longs, l´atteinte des os de la face et la mandibule représente 10 à12% des cas. Ce type de tumeur est extrêmement rare au niveau de la voûte du crâne, nous avons recueilli les 10 cas dans la littérature d´ostéoblastome confirmé de l´os pariétal sous forme de cas clinique. Dans cette série, l´âge des patients atteints d´ostéoblastome varient entre 5 et 57 ans, avec une moyenne d´âge de 22 ans, avec une incidence maximale durant les trois premières décennies de la vie. Nous avions 60% de patients de sexe féminin et 40% de sexe masculin [7,8]. Dans la majorité des cas, le mode de présentation clinique était sous la forme d´une tuméfaction indolore (50% des cas) du fait du caractère superficiel de cet os. Des cas de masses douloureuses de type inflammatoire ou mixtes avec des céphalées ont également été reportés [9]. Notre cas rapporté ici est le premier cas colligé dans notre service.

La sémiologie radiologique n´est pas spécifique [4,5,10], l´aspect scannographique habituel de l´ostéoblastome est une masse intramédullaire expansive, généralement bien délimitée d´une taille moyenne de 3 cm. Il montre également une destruction osseuse, une prise de contraste et parfois quelques calcifications. L´ostéoblastome est souvent plus vascularisé que l´ostéome ostéoïde et son aspect radiographique présente généralement des signes d´agressivité. L´aspect IRM est généralement hypo ou isointense en T1 et hyperintense en T2 avec un rehaussement variable à l´injection de gadolinium. Sur le plan histologique, l´aspect de nidus est caractéristique, avec la présence d´un réseau interconnecté de travées osseuses composées d´os lamellaire, disposées au sein d´un stroma lâche, richement vascularisé. Les cellules tumorales ne présentent pas de pléomorphisme nucléaire même si l´index mitotique peut être parfois élevé [5,6]. Parfois, il existe des aspects d´ostéoblastes proéminents avec des cellules géantes multinucléées de type ostéoclaste-like. Le traitement est essentiellement chirurgical, le risque de transformation maligne rare mais rapporté dans la littérature [11], confirme l´intérêt d´une résection totale avec une marge de sécurité sur de l´os sain chaque fois que c´est possible permet de prévenir aussi les récidives.

## Conclusion

L´ostéoblastome est considéré comme une tumeur bénigne, ostéoformatrice et bien circonscrite et à croissance rapide. Elle est rarement située au niveau de la calvaria, et affecte le plus souvent les sujets jeunes dans les 3 premières décennies de la vie. La traduction radio-clinique est non spécifique et Le diagnostic est histologique. Le traitement est chirurgical, Il nécessite chaque fois que possible, une exérèse totale pour éviter la récidive et la transformation maligne.
